# Supplementary material for: ClpP protease activation results from the reorganization of the electrostatic interaction networks at the entrance pores
Source: Commun Biol. 2019 Nov 13;2:410. doi: 10.1038/s42003-019-0656-3 (PMC6853987; doi:10.1038/s42003-019-0656-3)
Supplement: Supplementary file 1 — Supplementary Figures and Legends [file 42003_2019_656_MOESM1_ESM.pdf]

Supplementary Figure 1

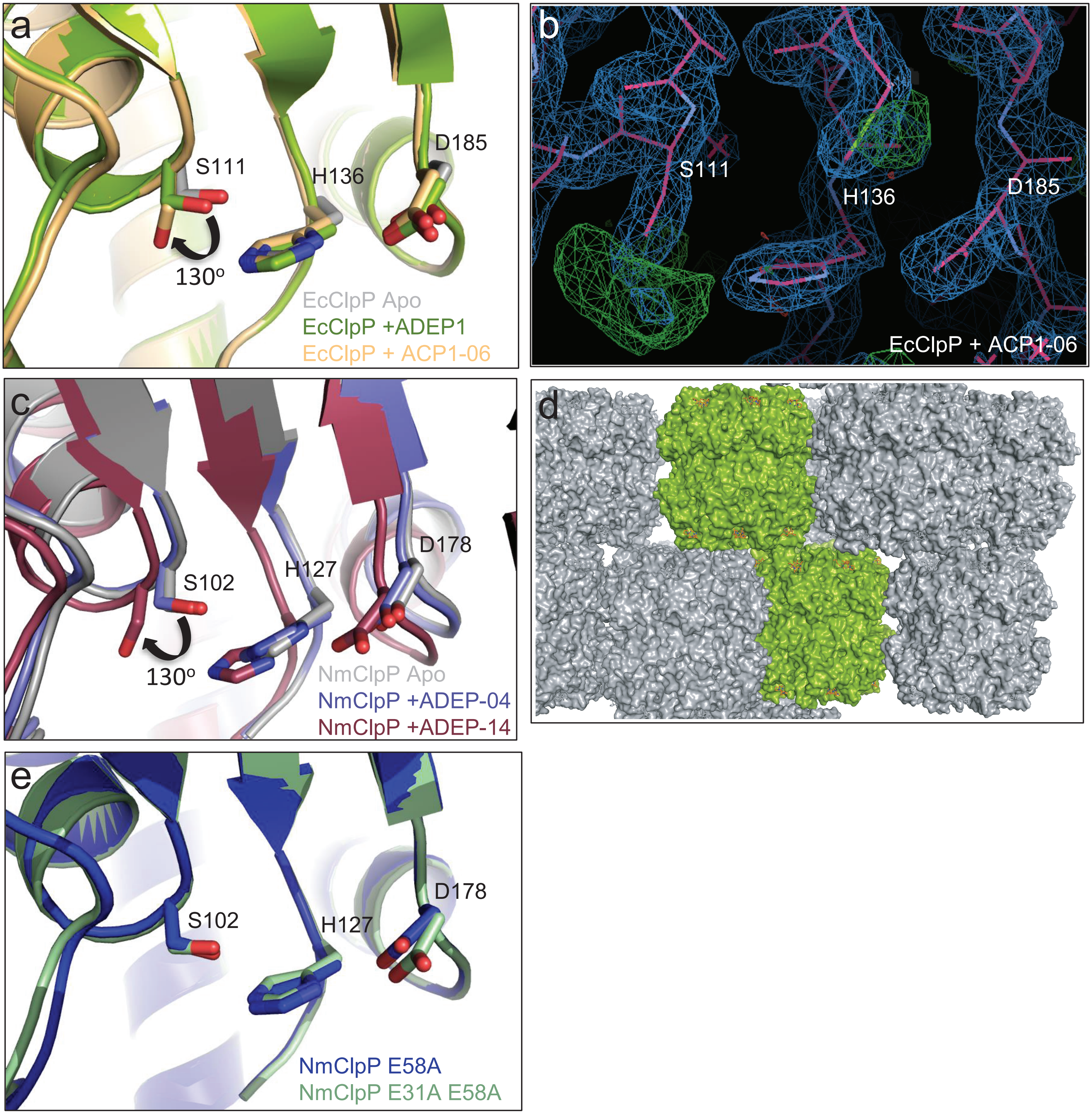

Supplementary Figure 1. Configuration of the Ser-His-Asp catalytic triad upon ClpP activation

(a) Superposition of apo-EcClpP, EcClpP+ADEP1, and EcClpP+ACP1-06 structures shows general preservation of the catalytic triad geometry, indicating that the structures represent active conformations of ClpP. In the presence of bound ACP1-06, the hydroxyl group of the catalytic Ser111 residue is rotated by ~130°, but still within hydrogen bonding distance to H136 for proton transfer during catalysis.

(b) $2F_o$-F_c (contoured at 1σ) and F_o-F_c (contoured at 3σ) electron density maps for the catalytic triad of EcClpP+ACP1-06, showing unresolved covalent modification of Ser111. This modification is not seen in activator-bound NmClpP structures.

(c) Binding of ADEP-04 and ADEP-14 to NmClpP did not perturb the geometry of the catalytic triad despite causing noticeable conformational change in the protein. In the NmClpP+ADEP-14 complex structure, the catalytic triad moves in the direction of the backbone shift but does not significantly affect the positions of the catalytic triad residues relative to each other.

(d) Axial loop ordering in the structure of NmClpP+ADEP-14 complex is prevented by crystal packing. The crystal asymmetric unit contains two tetradecamers of NmClpP (green surface).

(e) Superposition of the active sites of NmClpP E58A and NmClpP E31A+E58A mutants shows conservation of catalytic triad geometry relative to the apo-NmClpP (shown in c). Only subtle global conformational changes are observed in the $C\alpha$ backbones of these activated mutants.

Supplementary Figure 2

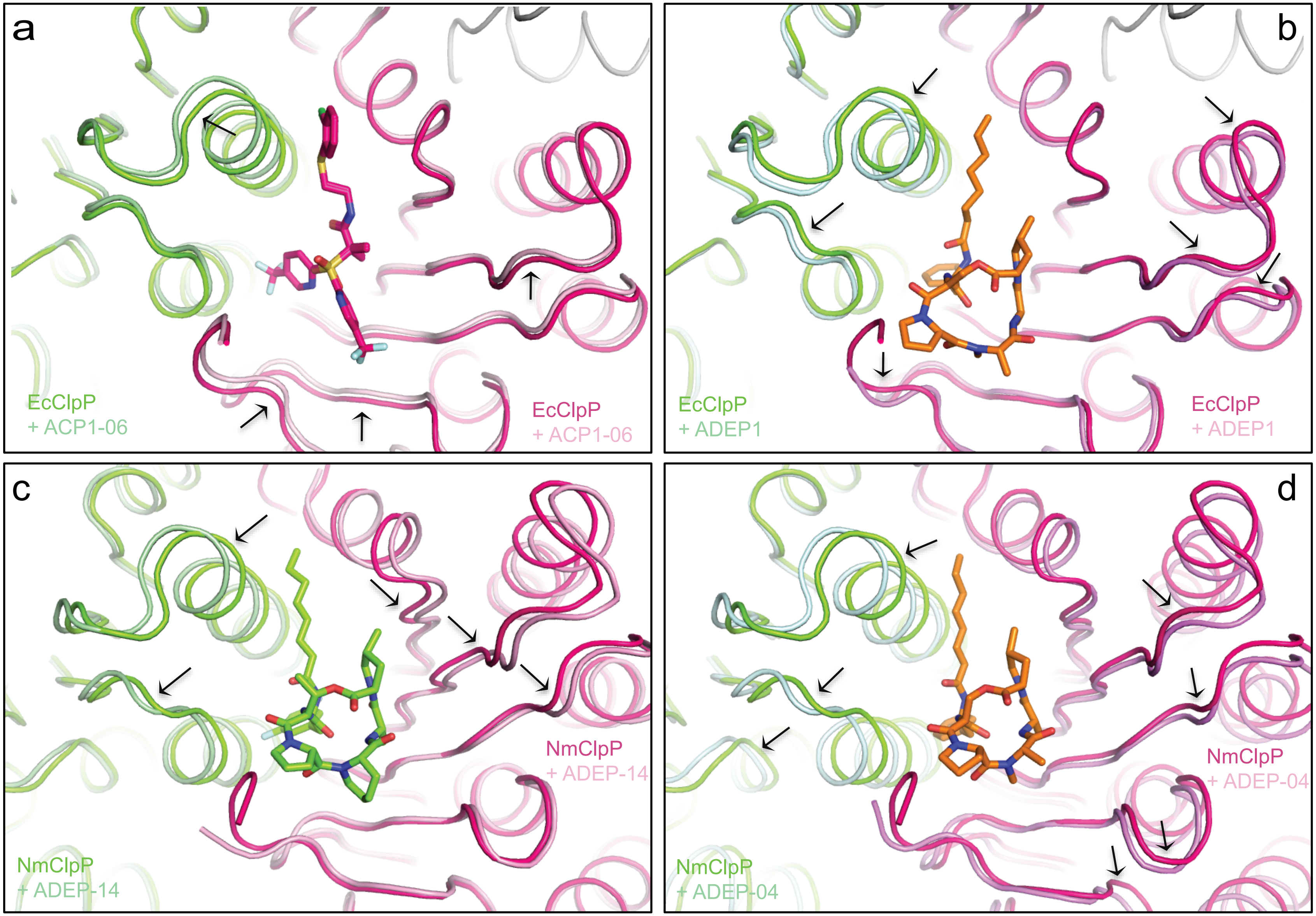

Supplementary Figure 2. Overlay of the apo- and compound-bound forms of EcClpP and NmClpP

(a,b) The panels show the movements of two neighboring subunits of EcClpP upon ACP1-06 and ADEP1 binding. ACP1-06 and ADEP1 move the ClpP subunits in different directions, as indicated by arrows. See Movies S1 and S2.

(c,d) The panels show the movements of two adjacent NmClpP subunits in the presence of bound ADEP-14 and ADEP-04. The effects of ADEP-14 and ADEP-04 binding to the ClpP Cα backbone is generally similar in directionality but not in magnitude as there are larger backbone shifts caused by ADEP-14. See Movies S3 and S4.

Supplementary Figure 3

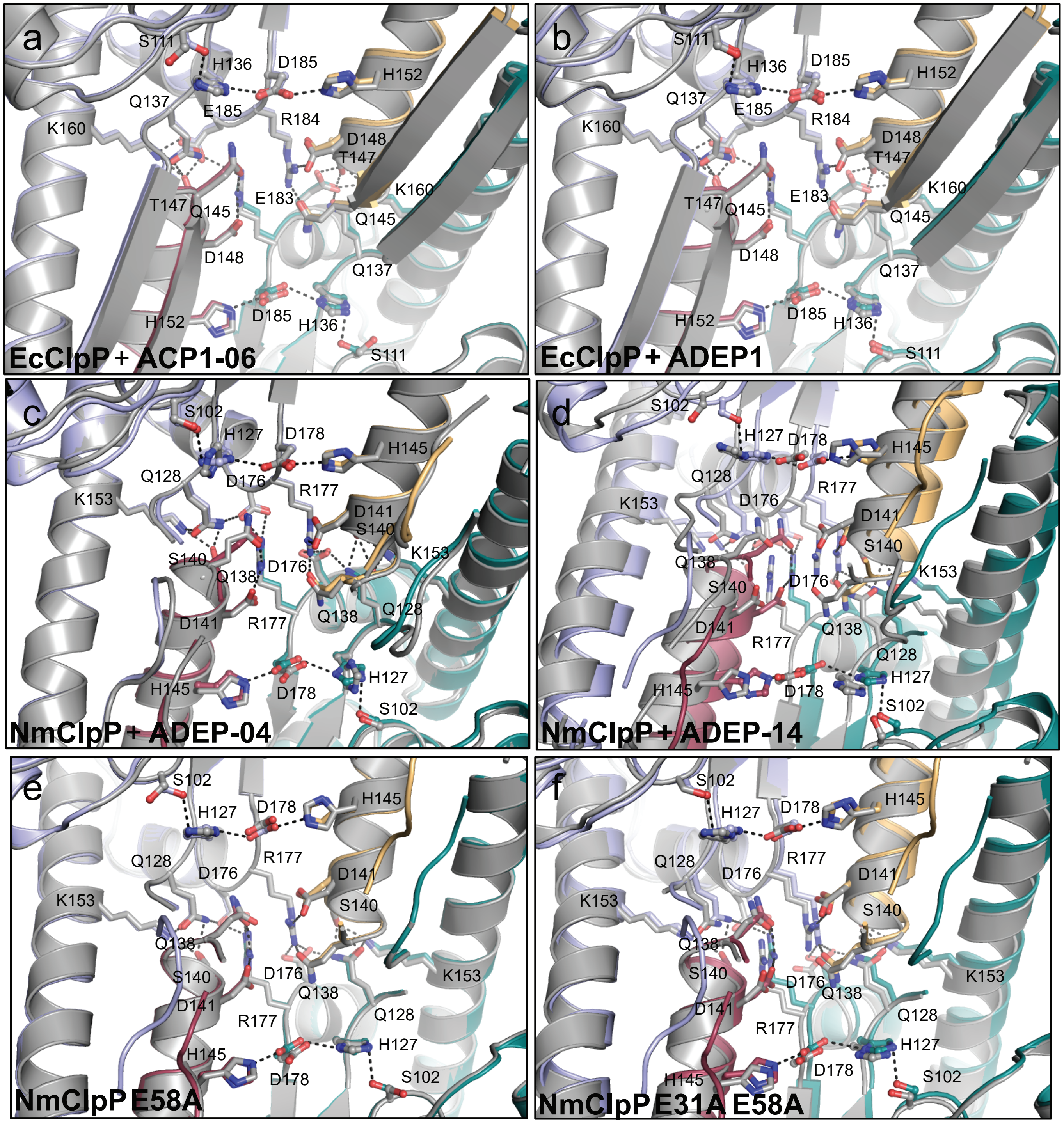

Supplementary Figure 3. Superposition of the equatorial regions in WT and activated ClpPs

(a,b) ACP1-06 and ADEP1 binding to EcClpP only cause minor shifts in the catalytic triad geometry, with ACP1-06 causing a repositioning of the S111 hydroxyl group that nevertheless remains catalytically competent. Here and in all panels, subunits of activated ClpP are shown in colored cartoon representations, while those of apo-ClpP are shown in gray.

(c,d) ADEP-04 and ADEP-14 binding show similar effects on the catalytic triad geometry in NmClpP without leading to a catalytically compromised conformation. ADEP-14 binding causes a more significant shift in the carbon backbones of the surrounding secondary structures than ADEP-04 binding, but the catalytic triad is moved in the general direction of the backbone shift, essentially preserving its geometry. In the NmClpP+ADEP-04 complex, the catalytic H127 residue rotates around the Cβ-Cγ bond, repositioning the imidazole group with respect to the side chains of S102 and D178.

(e,f). Activated mutants of NmClpP show preservation of the catalytic triad geometry relative to that of the wild-type enzyme.

Supplementary Figure 4

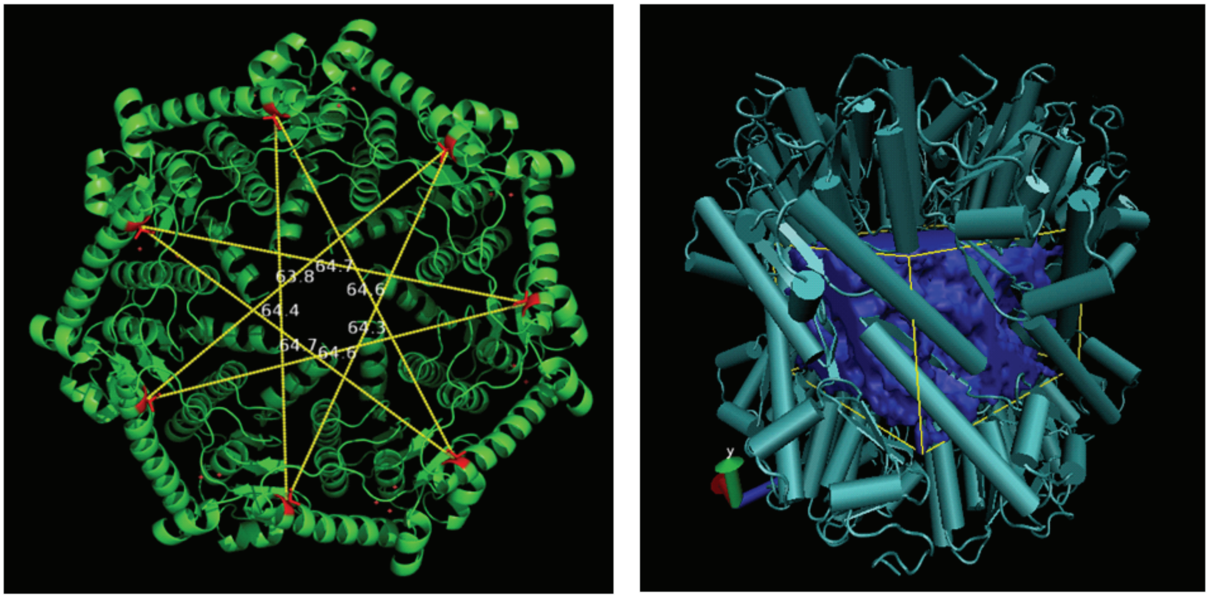

Structure	Mean distance (Å)	Catalytic chamber volume (Å^3)
NmClpP Apo (6NAQ)	63.5	75,000
NmClpP + ADEP-04 (5DKP)	62.5	73,300
NmClpP + ADEP-14 (6NAH)	61.0	72,500
NmClpP E58A (6NAW)	62.3	73,800
NmClpP E31A+E58A (6NAY)	62.8	74, 400
EcClpP Apo (1YG6)	65.4	66,000
EcClpP + ADEP1 (3MT6)	63.6	64,700
EcClpP + ACP1-06 (6NB1)	63.9	62,900
BsClpP Apo (3KTG)	62.8	68,200
BsClpP + ADEP1 (3KTI)	62.1	66,400
BsClpP + ADEP2 (3KTJ)	62.0	68,000
SaClpP Apo (3V5E)	62.4	66,600
SaClpP + ADEP (5VZ2)	62.1	66,300
MtClpP1P2 Apo (5DZK)	64.7	65,600
MtClpP1P2 + ADEP-2B^{5Me} (4U0G)	64.5	63,800

Supplementary Figure 4. Constriction of the equatorial region and decrease in the catalytic chamber volume of ClpP upon activation

The distances between two opposing Cα atoms of a conserved αE helix residue of the handle domain were measured as indicated to obtain the average diameter of the heptameric ClpP ring at the equator. The conserved residues used are I142 for NmClpP, I135 for EcClpP, I135 for BsClpP, I136 for SaClpP, and I136 for MtClpP1. Using the program VolArea[1], the catalytic chamber volumes of ClpP from *N. meningitidis*, *E. coli*, *B. subtilis*, *S. aureus*, and *M. tuberculosis* in the presence or absence of activating ligands or mutations, were measured and compared. Activated structures have smaller catalytic chambers compared to non-activated ones due to conformational changes in the tetradecameric structure.

Supplementary Figure 5

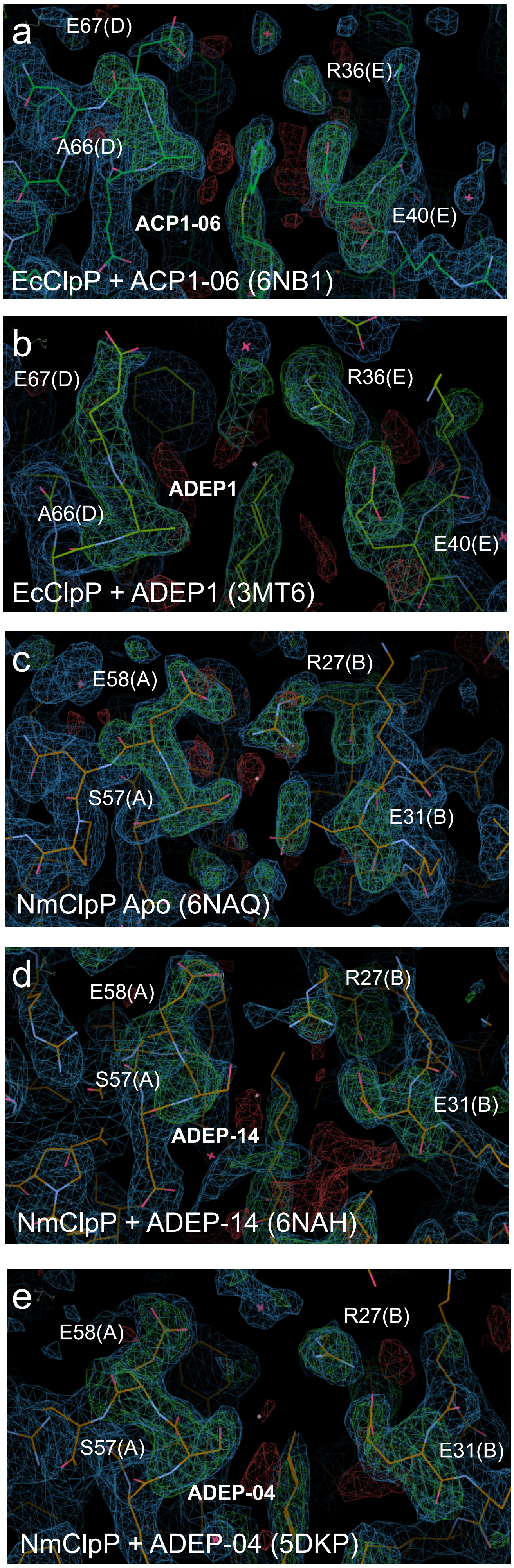

Supplementary Figure 5. Simulated annealing (SA) omit maps for residues involved in electrostatic interactions at the axial pore.

$2F_o$-F_c difference maps (blue) are contoured at 0.9σ and F_o-F_c omit maps are contoured at 3.5σ. Shown are SA omit maps for the four key residues involved in electrostatic interactions at the axial pore, corresponding to those in Figure 3b-f, respectively. The corresponding SA omit map for Figure 3a is not supplied because the reflection file used for calculating electron density maps is not available in the PDB.

Supplementary Figure 6

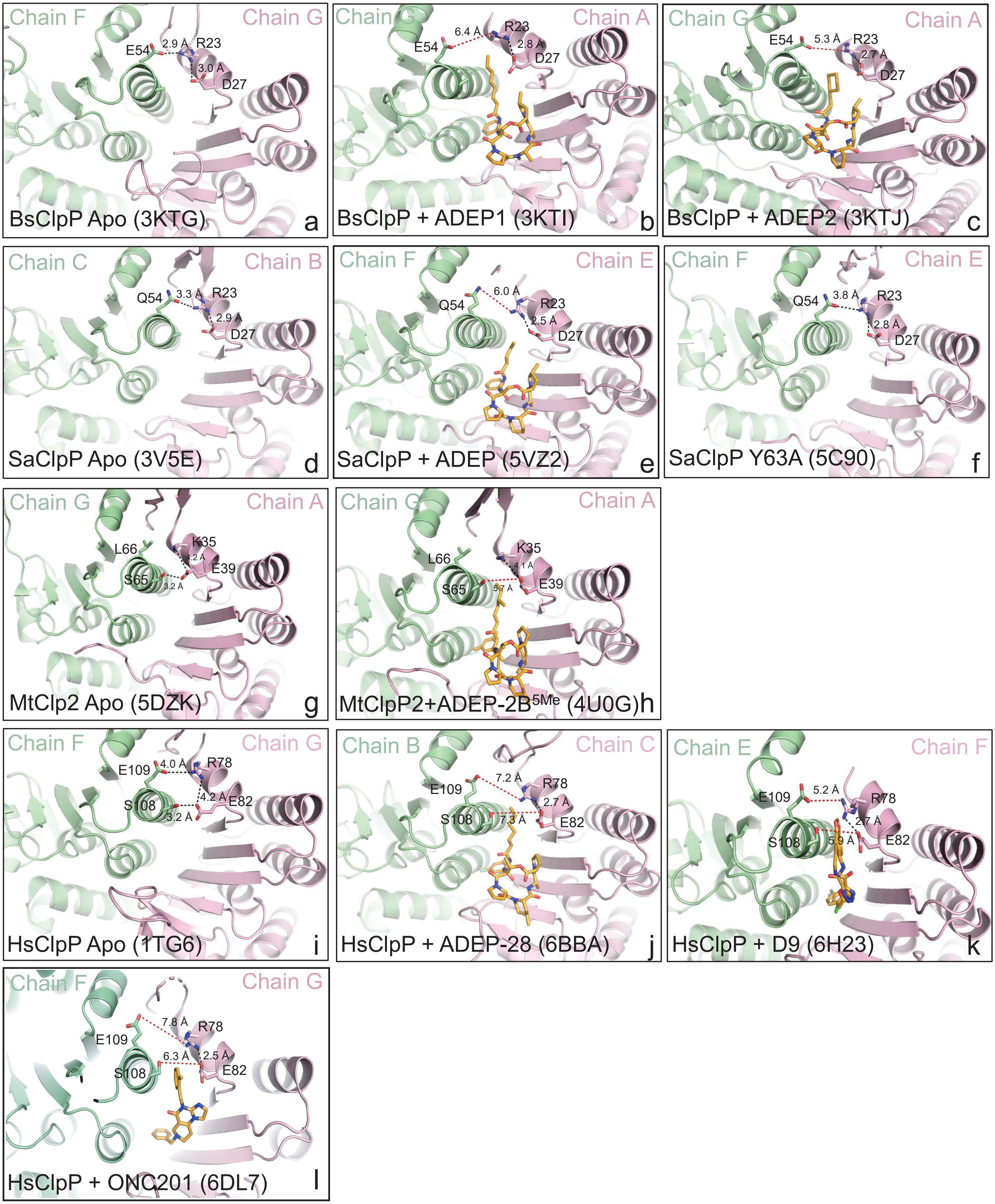

Supplementary Figure 6. Reorganization of non-covalent interactions near the axial pore upon ADEP binding or upon introduction of activating mutations

Structures of apo- and activated-ClpPs from *B. subtilis*, *S. aureus*, *M. tuberculosis*, and *Homo sapiens* are compared, with focus on the noncovalent bonding networks near the axial pore. Interactions discussed in the text are highlighted. If the atom distance is ≤ 4.3 Å then the dashed line is in black, otherwise the dashed line is in red.

Supplementary Figure 7

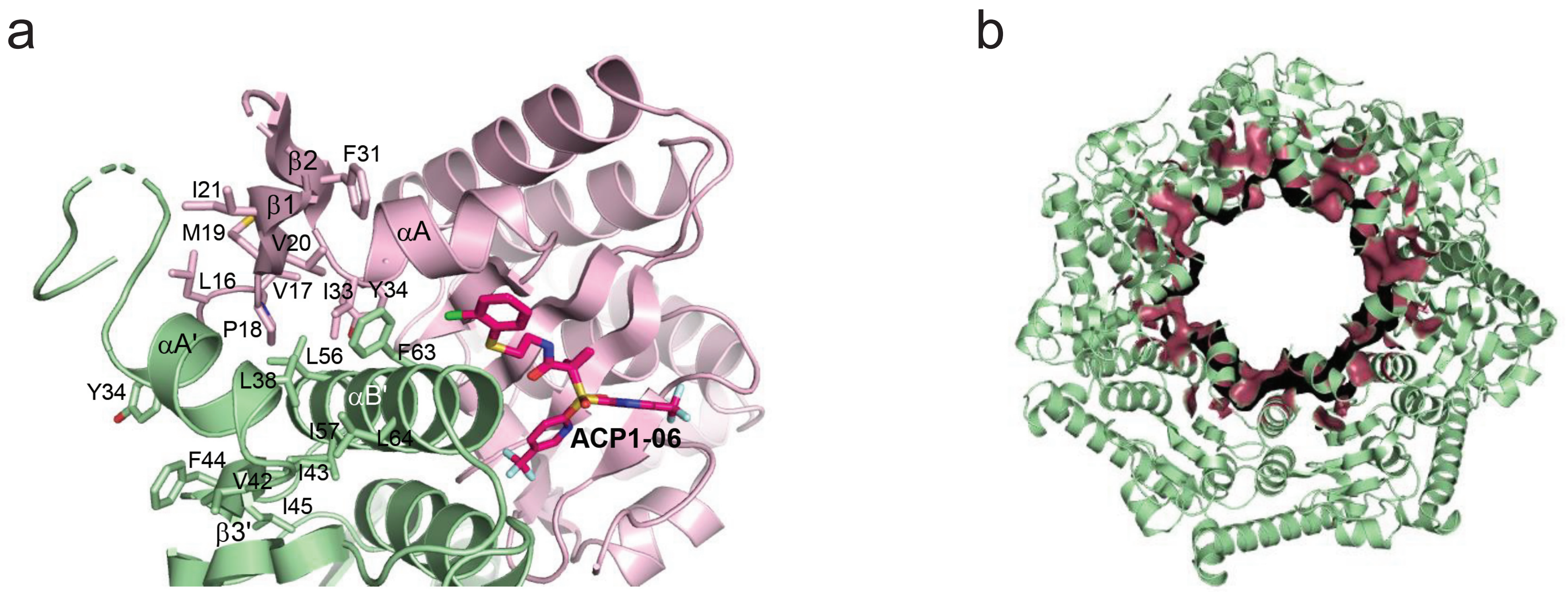

Supplementary Figure 7. Stabilization of ordered axial loops by hydrophobic interactions

(a) In the EcClpP+ACP1-06 structure, nonpolar residues in one subunit (in pink) at the N-terminus (L16, V17, P18) and on strand β1 (M19, V20, I21) interact with hydrophobic residues of nearby helices αA (I33, Y34, L38) and αB' (L56, I57, F63, L64; neighboring subunit in green) as well as strand β2 (F31). Hydrophobic residues on strand β3' (V42, I43, F44 and I45) also form part of the hydrophobic interaction surface.

(b) Shown is the continuous hydrophobic patch on the inner face of the axial pore of the EcClpP+ACP1-06 heptameric ring (red surface), formed by residues I33, Y34, L38, V42, I43, F44, L45, L56, I57, F63, and L64. This hydrophobic face facilitates the anchoring of the ordered N-terminal loops of ClpP.

Supplementary Figure 8

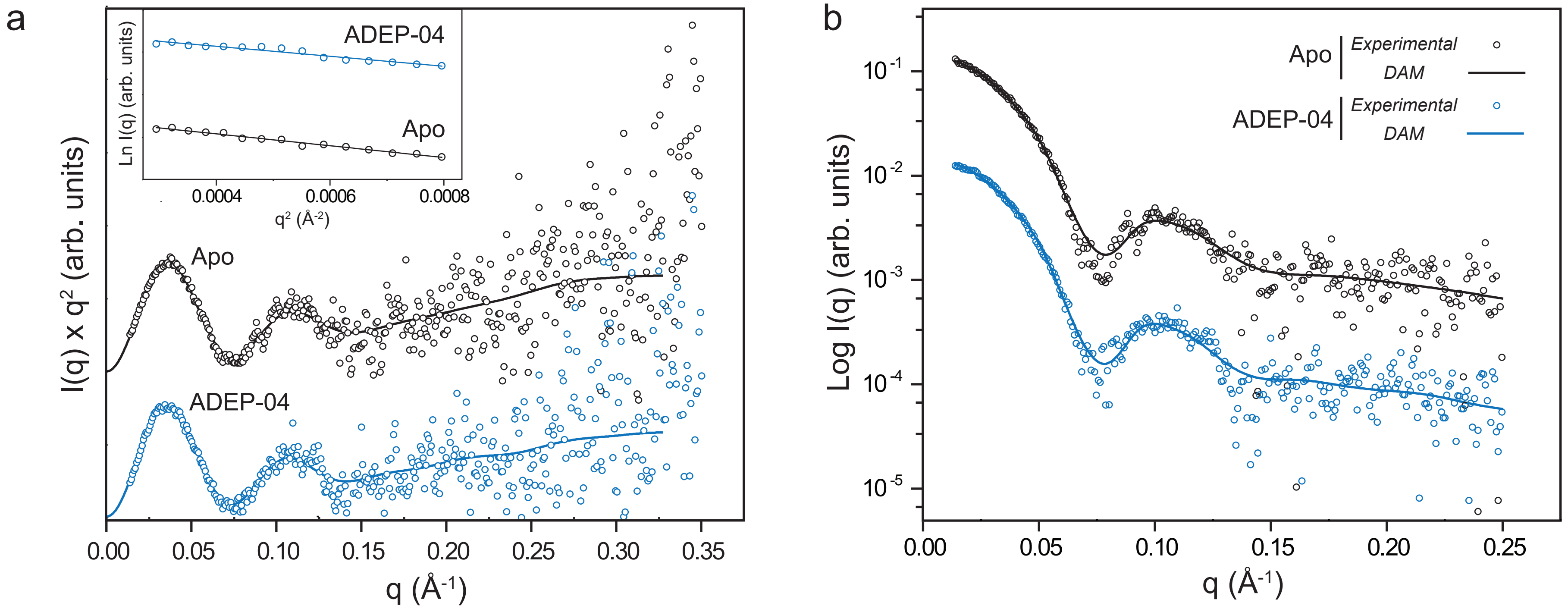

c

	Samples	Rg (Å) Guinier	Rg (Å) p(r)	Rg (Å) Hydropro	D_{max} (Å) p(r)	D_{max} (Å) Hydropro	MM (kDa)	Oligomeric state[c]
SAXS	NmClpP Apo	44 ± 2	44 ± 1	43[a]	122 ± 5	126[a]	321[b]	14.2
SAXS	NmClpP+ADEP-04	43 ± 2	44 ± 1	44[a]	115 ± 5	122[a]	319[b]	14.1

[a] Calculated using DAMs.

[b] Estimated using the final merged curve by the SAXS MoW program (http://saxs.ifsc.usp.br/).

[c] Calculated by dividing estimated MM values by the MM of NmClpP monomer (22.6 kDa).

Supplementary Figure 8. Small angle X-ray scattering data analysis and *ab initio* modeling of NmClpP

(a) Kratky analysis of NmClpP in the absence (black) and presence (blue) of ADEP-04. Experimental data are shown as symbols and GNOM curves as solid lines. *Inset:* Guinier approximation and linearity of the Guinier region of NmClpP samples. NmClpP+ADEP-04 curves were rescaled for comparison reasons.

(b) Fit between experimental and DAM models scattering curves of NmClpP. Black and blue circles represent experimental data; solid lines represent averaged simulated scattering curves of respective dummy atoms models (DAMs) generated by the DAMMIN program[2]. NmClpP+ADEP-04 curves were divided by 10 for visualization purposes.

(c) Size and dimension properties of apo-NmClpP and NmClpP+ADEP-04 determined by SAXS, and validation of DAMs using the Hydropro software.

Supplementary Figure 9

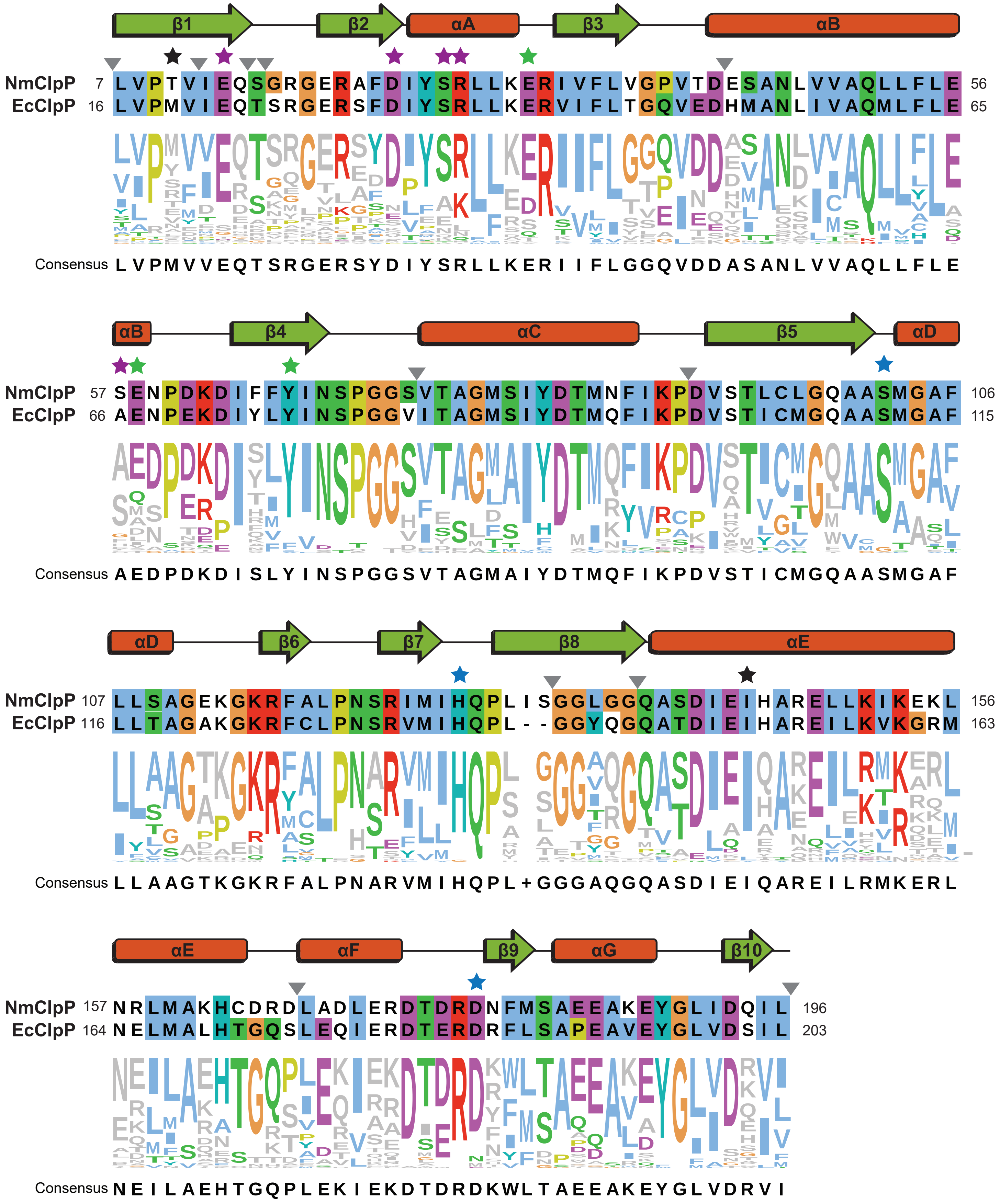

Supplementary Figure 9. Multiple sequence alignment of bacterial ClpPs

Multiple sequence alignment of 460 bacterial ClpP protein sequences (obtained from UniProt) was done using the Clustal Omega tool[3] with default parameters in JalView version 2.10.5[4]. The resulting alignment was displayed as a normalized consensus logo with all columns scaled to the same height to represent the relative amount of the residue per column by its size in the logo. The largest letter represents the modal residue in the column. Multi-modal values are not shown in the logo but are indicated by a "+" sign in the consensus sequence. Residues of the Ser-His-Asp catalytic triad are marked with blue stars. Sequences in the alignment that are not part of the NmClpP or EcClpP sequences have been removed and are indicated by a grey triangle. Residues mutated in NmClpP (E31, E58) and EcClpP (E40, E67, Y76) as described in Fig. 4a,b are marked with green stars. Residues that participate in electrostatic interactions near the axial pore are marked with purple stars (E13, D23, S26, R27 and S57 in NmClpP). Residue S57 in NmClpP corresponds to A66 in EcClpP. Residue T10 and I144 of NmClpP mutated in spin-labeling NMR studies are marked with black stars. Secondary structures of NmClpP are shown on top of the sequences.

REFERENCES

1. Ribeiro JV, Tamames JA, Cerqueira NM, Fernandes PA, Ramos MJ. Volarea - a bioinformatics tool to calculate the surface area and the volume of molecular systems. *Chem Biol Drug Des* **82**, 743-755 (2013).

2. Franke D, Svergun DI. DAMMIF, a program for rapid ab-initio shape determination in small-angle scattering. *J Appl Crystallogr* **42**, 342-346 (2009).

3. Madeira F, *et al.* The EMBL-EBI search and sequence analysis tools APIs in 2019. *Nucleic Acids Res* **47**, W636-W641 (2019).

4. Waterhouse AM, Procter JB, Martin DM, Clamp M, Barton GJ. Jalview Version 2--a multiple sequence alignment editor and analysis workbench. *Bioinformatics* **25**, 1189-1191 (2009).
