# Supplementary material for: ClpP protease activation results from the reorganization of the electrostatic interaction networks at the entrance pores
Source: Commun Biol. 2019 Nov 13;2:410. doi: 10.1038/s42003-019-0656-3 (PMC6853987; doi:10.1038/s42003-019-0656-3)
Supplement: Supplementary file 2 — Description of Additional Supplementary Files [file 42003_2019_656_MOESM2_ESM.docx]

**LEGENDS FOR SUPPLEMENTARY MOVIES**

**Supplementary Movie 1.** Conformational changes in EcClpP upon ACP1-06 binding. One crystal structure is of EcClpP in the apo-form. Binding of ACP1 (pink sticks) to the hydrophobic pockets causes the EcClpP cylinder to tighten by drawing all 14 subunits inwards towards the central vertical axis.

**Supplementary Movie 2.** Conformational changes in EcClpP upon ADEP1 binding. One crystal structure is EcClpP in the apo-form. Binding of ADEP1 (blue sticks) to the hydrophobic pockets causes significant structural changes including the ordering of the axial loops on the top and bottom pores, opening of the axial pore, and constriction of the equatorial region.

**Supplementary Movie 3.** Conformational changes in NmClpP upon ADEP-04 binding. One crystal structure is NmClpP in the apo-form. Binding of ADEP-04 (brown sticks) to the hydrophobic pockets causes the ordering of the axial loops, opening of the axial pores, and constriction of the equatorial region.

**Supplementary Movie 4.** Conformational changes in NmClpP upon ADEP-14 binding. One crystal structure is NmClpP in the apo-form. Binding of ADEP-14 (green sticks) to the hydrophobic pockets causes the opening of the axial pores and constriction of the equatorial region. The degree of conformational change caused by ADEP-14 binding is greater compared to that of ADEP-04 binding (Supplementary Movie 3). Axial loop ordering is not observed in the crystal structure due to crystal packing.

**Supplementary Movie 5.** Conformational changes in NmClpP in the presence of the activating mutation E58A. One crystal structure is NmClpP in the apo-form, while the other is that of the NmClpP E58A mutant. The conformational change induced by mutation is similar to but less pronounced than that of ADEP-binding to NmClpP (Supplementary Movies 3 and 4), characterized by axial pore opening and constriction at the equatorial region. Axial loop ordering is not observed in the NmClpP E58A mutant structure due to crystal packing.

**Supplementary Movie 6.** Conformational changes in NmClpP in the presence of the activating mutations E31A and E58A. The first crystal structure is NmClpP in the apo-form, followed by the NmClpP E31A+E58A double mutant. The conformational change induced by mutation is similar to but less pronounced than that of ADEP-binding to NmClpP (Supplementary Movies 3 and 4), characterized by axial pore opening and constriction at the equatorial region. As in the NmClpP E58A mutant structure, axial loop ordering is not observed in the NmClpP E31A+E58A double mutant structure due to crystal packing.

**LEGENDS FOR SUPPLEMENTARY DATA**

**Supplementary Data 1.** Source data for Fig. 4a,b.
