# Supplementary figures and images for: ClpP protease activation results from the reorganization of the electrostatic interaction networks at the entrance pores
Source: Commun Biol. 2019 Nov 13;2:410. doi: 10.1038/s42003-019-0656-3 (PMC6853987; doi:10.1038/s42003-019-0656-3)

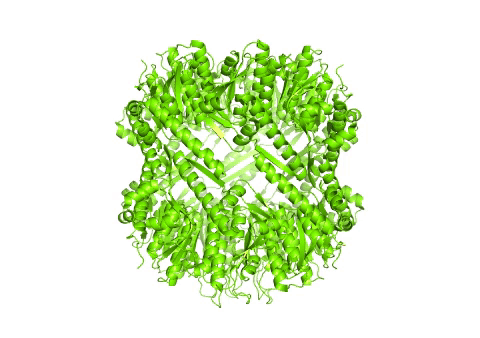

Supplement: Supplementary file 4 — Supplementary Movie 1 [file 42003_2019_656_MOESM4_ESM.gif]

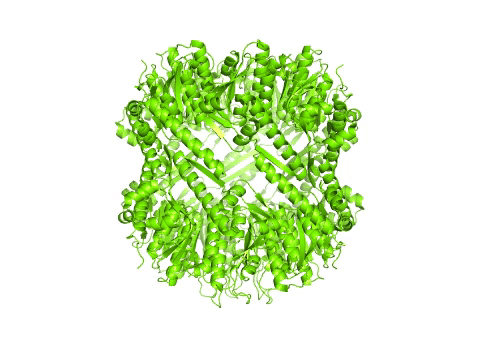

Supplement: Supplementary file 5 — Supplementary Movie 2 [file 42003_2019_656_MOESM5_ESM.gif]

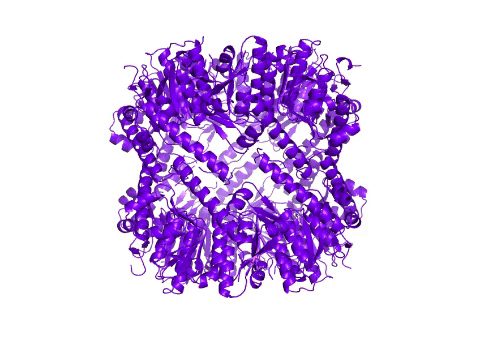

Supplement: Supplementary file 6 — Supplementary Movie 3 [file 42003_2019_656_MOESM6_ESM.gif]

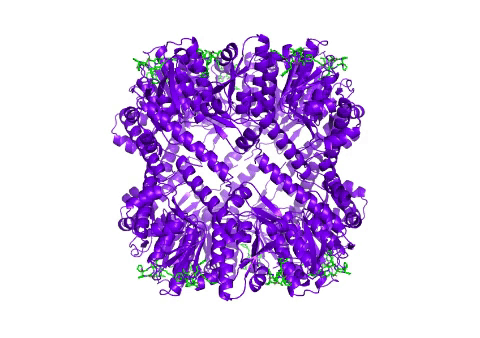

Supplement: Supplementary file 7 — Supplementary Movie 4 [file 42003_2019_656_MOESM7_ESM.gif]

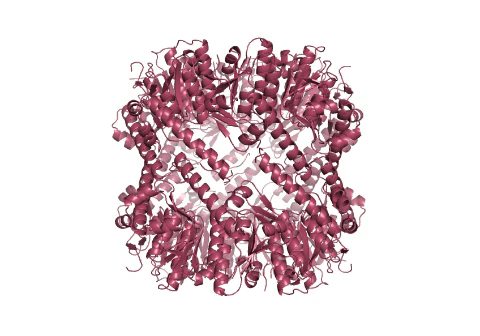

Supplement: Supplementary file 8 — Supplementary Movie 5 [file 42003_2019_656_MOESM8_ESM.gif]

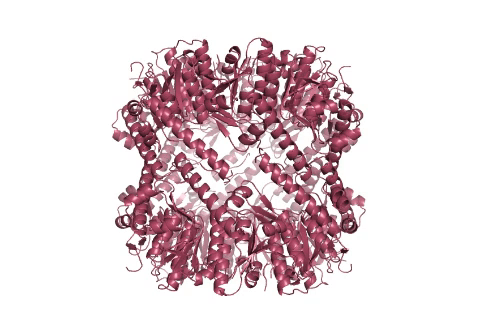

Supplement: Supplementary file 9 — Supplementary Movie 6 [file 42003_2019_656_MOESM9_ESM.gif]
