# Supplementary material for: ClpP protease activation results from the reorganization of the electrostatic interaction networks at the entrance pores
Source: Commun Biol. 2019 Nov 13;2:410. doi: 10.1038/s42003-019-0656-3 (PMC6853987; doi:10.1038/s42003-019-0656-3)
Supplement: Supplementary file 10 — Reporting Summary [file 42003_2019_656_MOESM10_ESM.pdf]

nature research

Corresponding author(s): Walid A. Houry

Last updated by author(s): Oct 11, 2019

Reporting Summary

Nature Research wishes to improve the reproducibility of the work that we publish. This form provides structure for consistency and transparency in reporting. For further information on Nature Research policies, see Authors & Referees and the Editorial Policy Checklist.

Statistics

For all statistical analyses, confirm that the following items are present in the figure legend, table legend, main text, or Methods section.

n/a	Confirmed	
☐	☒	The exact sample size (*n*) for each experimental group/condition, given as a discrete number and unit of measurement
☐	☒	A statement on whether measurements were taken from distinct samples or whether the same sample was measured repeatedly
☒	☐	The statistical test(s) used AND whether they are one- or two-sided<br>*Only common tests should be described solely by name; describe more complex techniques in the Methods section.*
☒	☐	A description of all covariates tested
☒	☐	A description of any assumptions or corrections, such as tests of normality and adjustment for multiple comparisons
☐	☒	A full description of the statistical parameters including central tendency (e.g. means) or other basic estimates (e.g. regression coefficient) AND variation (e.g. standard deviation) or associated estimates of uncertainty (e.g. confidence intervals)
☒	☐	For null hypothesis testing, the test statistic (e.g. *F*, *t*, *r*) with confidence intervals, effect sizes, degrees of freedom and *P* value noted<br>*Give P values as exact values whenever suitable.*
☒	☐	For Bayesian analysis, information on the choice of priors and Markov chain Monte Carlo settings
☒	☐	For hierarchical and complex designs, identification of the appropriate level for tests and full reporting of outcomes
☒	☐	Estimates of effect sizes (e.g. Cohen's *d*, Pearson's *r*), indicating how they were calculated

Our web collection on statistics for biologists contains articles on many of the points above.

Software and code

Policy information about availability of computer code

Data collection	Macromolecular Crystallography Data Collector software (MxDC) for remote data collection at the Canadian Light Source, JDirector v 2.2.1 r8 for data collection at the Structural Genomics Consortium at the University of Toronto, TopSpin (Bruker) for NMR
Data analysis	HKL2000, Mosflm, Scala, Xprep, Shelx, PHENIX, ATSAS 2.7.2 software package (DATMERGE, GNOM, DAMMIN, DAMAVER), SAXSMoW, UCSF Chimera, NMRPipe, CcpNMR

For manuscripts utilizing custom algorithms or software that are central to the research but not yet described in published literature, software must be made available to editors/reviewers. We strongly encourage code deposition in a community repository (e.g. GitHub). See the Nature Research guidelines for submitting code & software for further information.

Data

Policy information about availability of data

All manuscripts must include a data availability statement. This statement should provide the following information, where applicable:

- Accession codes, unique identifiers, or web links for publicly available datasets
- A list of figures that have associated raw data
- A description of any restrictions on data availability

The new protein structures presented in this study are freely available in the Protein Data Bank with accession codes 6NB1, 6NAQ, 6NAH, 6NAW, and 6NAY.

Field-specific reporting

Please select the one below that is the best fit for your research. If you are not sure, read the appropriate sections before making your selection.

☒ Life sciences ☐ Behavioural & social sciences ☐ Ecological, evolutionary & environmental sciences

Life sciences study design

All studies must disclose on these points even when the disclosure is negative.

Sample size	During refinement of all protein structures, the Rfree set is generated automatically by Phenix and includes 5% of the total number of reflections or 2,000 reflections, whichever is lower. The Rwork set is the rest of the reflections without the Rfree set. For small angle X-ray scattering, data were collected using 10 x 10 sec and 1 x 300 sec X-ray exposure times. After comparison, the curves were merged to give rise to a single averaged X-ray scattering profile. Ab initio models are average of ten independent runs. Distance measurements were repeated 3 times for each generated ab initio model.
Data exclusions	*Describe any data exclusions. If no data were excluded from the analyses, state so OR if data were excluded, describe the exclusions and the rationale behind them, indicating whether exclusion criteria were pre-established.*
Replication	All enzymatic assays of ClpP were done in triplicate.
Randomization	During refinement of all protein structures, the Rfree set is generated automatically by Phenix and includes 5% of the total number of reflections or 2,000 reflections, whichever is lower. For SAXS, ten ab initio models were independently generated and compared by means of normalized spatial discrepancy by DAMAVER.
Blinding	*Describe whether the investigators were blinded to group allocation during data collection and/or analysis. If blinding was not possible, describe why OR explain why blinding was not relevant to your study.*

Reporting for specific materials, systems and methods

We require information from authors about some types of materials, experimental systems and methods used in many studies. Here, indicate whether each material, system or method listed is relevant to your study. If you are not sure if a list item applies to your research, read the appropriate section before selecting a response.

Materials & experimental systems

n/a	Involved in the study	
☒	☐	Antibodies
☒	☐	Eukaryotic cell lines
☒	☐	Palaeontology
☒	☐	Animals and other organisms
☒	☐	Human research participants
☒	☐	Clinical data

Methods

n/a	Involved in the study	
☒	☐	ChIP-seq
☒	☐	Flow cytometry
☒	☐	MRI-based neuroimaging
